# Amphotericin B and Other Polyenes—Discovery, Clinical Use, Mode of Action and Drug Resistance

**DOI:** 10.3390/jof6040321

**Published:** 2020-11-27

**Authors:** Hans Carolus, Siebe Pierson, Katrien Lagrou, Patrick Van Dijck

**Affiliations:** 1 VIB-KU Leuven Center for Microbiology, 3001 Leuven, Belgium; hans.carolus@kuleuven.be (H.C.); siebe.pierson@kuleuven.be (S.P.); 2 Laboratory of Molecular Cell Biology, Department of Biology, KU Leuven, 3001 Leuven, Belgium; 3 Laboratory of Clinical Bacteriology and Mycology, Department of Microbiology, Immunology and Transplantation, KU Leuven, 3001 Leuven, Belgium; katrien.lagrou@uzleuven.be; 4 Department of Laboratory Medicine and National Reference Center for Mycosis, UZ Leuven, 3001 Leuven, Belgium

**Keywords:** amphotericin B, polyene, antifungal drug resistance, mode of action, discovery

## Abstract

Although polyenes were the first broad spectrum antifungal drugs on the market, after 70 years they are still the gold standard to treat a variety of fungal infections. Polyenes such as amphotericin B have a controversial image. They are the antifungal drug class with the broadest spectrum, resistance development is still relatively rare and fungicidal properties are extensive. Yet, they come with a significant host toxicity that limits their use. Relatively recently, the mode of action of polyenes has been revised, new mechanisms of drug resistance were discovered and emergent polyene resistant species such as *Candida auris* entered the picture. This review provides a short description of the history and clinical use of polyenes, and focusses on the ongoing debate concerning their mode of action, the diversity of resistance mechanisms discovered to date and the most recent trends in polyene resistance development.

## 1. History of Polyenes as Antifungal Drugs

**Figure 1:**
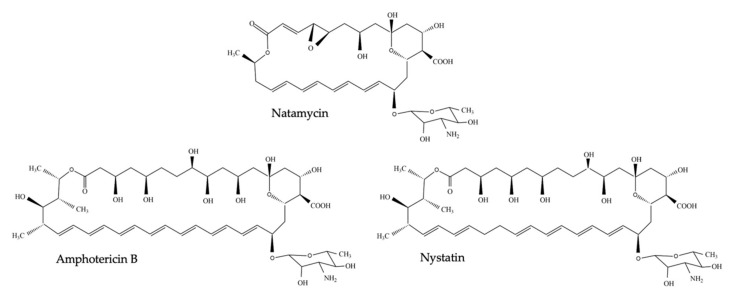
Chemical structure of nystatin, amphotericin B and natamycin (pimaricin).

Polyenes were, after griseofulvin [1], the first fungal-specific antibiotics on the market and ever since, more than 200 polyene antifungals have been discovered [2], of which amphotericin B, nystatin and natamycin (Figure 1) are most commonly used in antifungal therapy [3]. In 1949, microbiologist Elizabeth L. Hazen and chemist Rachel F. Brown isolated the first antifungal polyene—fungicidin—later called nystatin after New York State, where the meeting of the National Academy of Sciences in which fungicidin was presented, took place that year [4,5,6]. Nystatin was purified as a fermentation product of *Streptomyces noursei,* cultured from a soil sample of the farm of W. Nourses, after which the antibiotic producing actinomycete species was named [6]. Nystatin was patented by the E.R. Squibb and Sons Institute and became one of the first antimycotic drugs on the market. Early on, it became apparent, however, that nystatin had poor gastrointestinal absorption and could thus only be used to treat topical mycoses [7]. Therefore, the same research group continued their broad screening of soil-cultivated fermentation broths and in January 1953, a fermentation broth from the *Streptomyces nodosus* culture M4575, cultured from a soil sample of the Orinoco basin in Venezuela, showed remarkable antifungal activity [5,7]. Two active compounds were isolated: amphotericin A and amphotericin B (AmB), named after their amphoteric properties. These polyenes were chemically similar to nystatin, but the ultraviolet absorption spectrum showed additional maxima at longer wavelengths [7]. After successful purification, the tetraene amphotericin A showed an antifungal spectrum similar to nystatin, while the heptaene AmB had a significantly greater antifungal activity compared to nystatin and amphotericin A [7]. It took over one and a half decades to completely unravel the chemical structure of AmB [8]. AmB is, like other polyenes, a complex macrolide antibiotic, characterized by an almost flat macrolactone ring (hence the name macrolide) with a series of conjugated double bonds (Figure 1) [9]. The latter discriminates polyenes from antibacterial macrolides such as erythromycin [9]. Depending on the number of conjugated double bonds, polyenes can be classified into trienes, tetraenes, pentaenes, hexaenes, heptaenes, etc. [9,10]. In general, polyenes consist of a hydrophobic polyene “tail” and a hydrophilic ”head” with a mycosamine group and a polyol chain that holds a number of hydroxyl groups [2].

Due to its amphipathic character, AmB is poorly water soluble and although initially oral treatment of infected mice seemed successful [7], no such effect was obtained in humans [6,7]. Eventually, researchers at the E.R. Squibb and Sons institute used a formulation in which AmB and sodium deoxycholate formed a micellar suspension when reconstituted in a glucose solution [6,7]. This preparation, named Fungizone^®^ and commonly referred to as AmB deoxycholate, could yield high blood concentrations of AmB upon intravenous administration and was very effective against systemic cryptococcosis, histoplasmosis and other deep mucosal infections [7]. Ever since the discovery of AmB, multiple different derivates and formulations have been developed. The latter to counteract the dose-limiting toxicities of AmB, which comprise nephrotoxicity and infusion-related complications (discussed in Section 2. Clinical Use) [11]. Nevertheless, AmB can still be regarded as one of the most fungicidal drugs on the market with a broad spectrum and limited incidence of resistance development (discussed in Section 4. Polyene Resistance), largely due to their mode of action (discussed in Section 3. Mode of Action) [2]. Today, polyenes are still regularly used beyond antifungal treatment, such as the AmB treatment of visceral leishmaniasis [12].

## 2. Strengths and Drawbacks of Polyene Use in the Clinic

Clinical use of AmB and other polyenes has extensively been reviewed elsewhere [2,11,13,14,15]; here, we only provide a short summary on the most common applications and drawbacks of the use of polyenes as antifungal drugs. About six polyene antifungals have been used for antifungal therapy: AmB, nystatin, natamycin (also called pimaricin), candicidin, trichomycin and methyl partricin [2]. However, only three polyenes remain in widespread therapeutic use today: AmB for systemic mycoses, nystatin for mucosal infections such as oral or vulvovaginal candidiasis and natamycin for ophthalmic infection [2,3,13]. Polyenes are still in use because of their broad spectrum of activity (cf. echinocandins, 1st and 2nd generation azoles) against pathogenic yeasts and molds, including *Candida* spp., *Aspergillus* spp., *Cryptococcus* spp., *Fusarium* spp., Mucorales (e.g., *Rhizopus* spp.) and endemic mycosis (e.g., *Histoplasma* spp.) [13]. Nevertheless, several studies show that AmB treatment of systemic mycoses caused by species such as *Aspergillus terreus* [16], *Scedosporium* spp. [17] and *Candida auris* [18] might not always be successful, often due to intrinsic or acquired resistance [14]. Still, polyene resistance is rarer [13,19,20], and the relative decrease in susceptibility is smaller, compared to resistance to other drug antifungal classes such as azoles or echinocandins [21,22] (see Section 4. Polyene Resistance). Nevertheless, due to its toxicity and the availability of the (tri)azole and echinocandin antifungals, the use of AmB to treat the most common systemic mycoses such as candidiasis and aspergillosis has decreased [13]. According to the Infectious Diseases Society of America (IDSA) [23] and European Confederation of Medical Mycology [24] (ECMM) guidelines, AmB is still recommended as first line treatment for severe cryptococcosis (often in combination with flucytosine), disseminated histoplasmosis and mucormycosis, while it remains an alternative for other infections upon intolerance, limited availability or failure of other treatments [13,23]. Furthermore, AmB has been recommended as a prophylaxis for invasive *Candida* [23] and *Aspergillus* [25] infections in solid-organ transplant recipients and patients receiving immunosuppressive treatment, respectively.

The biggest constraint concerning the use of AmB in the clinic is its intrinsic host toxicity. This dose-related toxicity limits the maximum tolerated dose to for example 0.7–1.0 mg/kg/day for AmB deoxycholate, which may be suboptimal to acquire clinical success [11]. Although the affinity of AmB to fungal ergosterol is over ten-fold higher compared to mammalian cholesterol, non-selective disruption of mammalian cell membranes does occur [26]. Renal toxicity and acute infusion-related adverse effects such as fever and nausea are most commonly associated with intravenous AmB administrations, while liver damage occurs but is less common [13]. Acute infusion-related toxicity is due to the fact that AmB, a molecule of microbial origin, is recognized by TLR2 (Toll-like receptor 2) and CD14 on mononuclear immune cells, leading to the initiation of an inflammatory response [11]. Nephrotoxicity is thought to be caused by increased exposure of AmB to renal cells via low-density lipoprotein (LDL) receptor mediated endocytosis. Moreover, AmB causes vasoconstriction in afferent renal arterioles, which decreases renal blood flow and glomerular filtration [11]. Lipid-associated AmB formulations, such as AmB lipid complex (ABLC) and liposomal AmB (L-AmB), have been developed with the main goal of improving the therapeutic index and reducing toxic complications compared to conventional AmB deoxycholate [11]. The pharmacokinetic parameters for these formulations differ substantially. For example, ABLC is large and taken up rapidly by macrophages in tissues such as the liver, spleen and lungs, while L-AmB is small and negatively charged, resulting in higher peak plasma levels compared to other formulations [11]. The effects of these lipid formulations on the clinical success and mortality of the patient are a subject of debate, and largely depend on the study, varying for type of infection (e.g., cryptococcosis and histoplasmosis) and background of the patients (e.g., AIDS and neutropenic) [11]. Overall, lipid AmB formulations show less nephrotoxicity compared to AmB deoxycholate, while L-AmB also exhibits less infusion-related reactions [11]. Still, new structures and formulations are developed to optimize the use of polyenes in the clinic. A recent example is the discovery of amphamide, an amide of AmB and termed a “second generation polyene antifungal” [27]. Amphamide was developed to increase the water solubility of AmB and shows an over 20-fold higher therapeutic index compared to AmB. Additionally, it has a superior antifungal activity and lower acute host toxicity in vivo [27]. Nanotechnology-based formulations of polyenes have also been investigated, aimed at decreasing their toxicity and/or increase their solubility, therapeutic index and/or oral availability. Nanoformulations include nanocrystals, nanotubes, polymeric nanocarriers, cubosomal and cochleate nanoparticles [28]. Although the low solubility and gastrointestinal absorption initially redeemed polyenes as oral drugs, recent research such as nanobody delivery shows great potential for the future [28,29].

## 3. Mode of Action of Polyene Antifungal Drugs

### 3.1. Polyene—Sterol Interactions

Overall, polyenes have an unusual mode of action compared to other antifungal drug classes, as they do not target a specific enzyme but rather interact with a vital molecule—ergosterol [1]. The first indications of the mode of action of polyenes were published in 1958, when Gottlieb et al. [30] discovered that the addition of sterols such as cholesterol, lanosterol and ergosterol could inhibit the fungicidal effect of nine polyenes including filipin, AmB and nystatin on three fungal species. They suggested that polyenes could prevent the synthesis of sterols (as the, then not yet discovered, azole, allylamine and phenylmorpholine antifungals do [1]) or competitively replace the sterols as a cofactor of an essential metabolic reaction [30]. Later, however, it became apparent that polyenes can alter the permeability of the membrane by reacting with sterols [10]. How this process of sterol sequestration works, remains subject to debate. The most studied mechanism of action is the pore forming model (see Section 3.2. Pore Forming Models) in which polyenes interact with ergosterol to form ion-leaking pores in the membrane [19]. Nevertheless, it has been shown that pore formation can also be established in the absence of sterols [31]. Early on, Cotero et al. [31] proposed that sterols have an essential role in the structure of the membrane itself during amphotericin activity, but might not be directly involved in the pore formation [31]. Later, other studies supported this idea [32,33,34,35,36,37]. For example, the polyene natamycin was shown to bind ergosterol without altering the cell membrane permeability [37]. Further research pointed out that natamycin inhibits various ergosterol-dependent membrane proteins and so disturbs essential cellular processes such as glucose transport, amino acid transport [38] and vacuolar fusion [36]. Currently, four models of the polyene mode of antifungal action have been proposed: the pore forming model, the surface adsorption model, the sterol sponge model and the oxidative damage model [39] (see Section 3.2 Pore Forming Models, Section 3.3 Surface Adsorption and Sterol Sponge Models and Section 3.4 Other Modes of Action).

**Figure 2:**
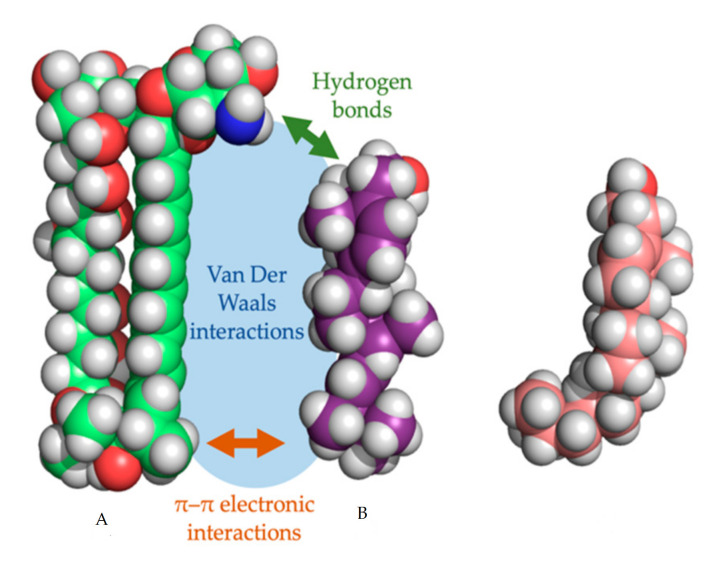
Three-dimensional model of amphotericin B (**a**) with the cylindrical ergosterol (**b**) and sigmoidal cholesterol (**c**). The three types of non-covalent interactions between amphotericin B and ergosterol are shown.

In every proposed model, the binding of the polyene with ergosterol is key to its antifungal effect [39]. Ergosterol plays an essential role in many cellular processes of fungi, including regulation of membrane proteins, endocytosis, cell division, membrane fluidity and cell signaling [40,41,42]. The specificity of therapeutic polyenes to ergosterol comes from the fact that ergosterol has a significantly different three-dimensional structure compared to mammalian cholesterol, enabling better binding into the hydrophobic ”pocket” of polyenes such as AmB, as depicted in Figure 2 [1]. Three interactive forces play a role in the binding of AmB and ergosterol: Van der Waals powers which are highest when both molecules are orientated co-planar and parallel, a hydrogen bond network between the 3β-OH group of the sterol and the polar mycosamine group of AmB and π–π electronic interactions between the ergosterol side chain and the polyene “tail” of AmB [43]. The latter essential “attach point” does not occur when AmB binds to cholesterol [39,43]. Moreover, Van Der Waals interactions are weaker between cholesterol and AmB due to the sigmoidal conformation of the sterol side-chain [39]. The specific binding, along with the higher ergosterol:phospholipid ratio in fungal cell membranes, compared to the cholesterol:phospholipid ratio in mammalian cells, explains the selectivity of most polyenes to fungal cells [1].

### 3.2. Pore Forming Models

**Figure 3:**
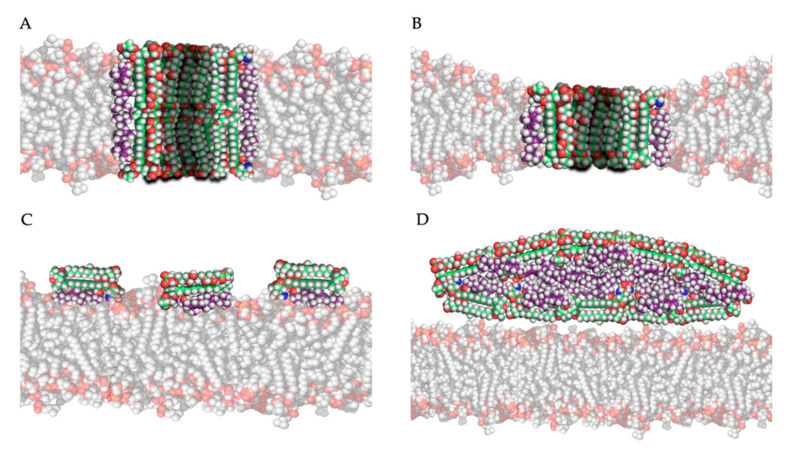
Four mechanistic models of the interaction of amphotericin B with ergosterol in/near the plasma membrane: (**A**) the pore forming model, (**B**) the half-pore forming model, (**C**) the surface adsorption model and (**D**) the sterol sponge model (Legend: see Figure 2).

The most studied model for polyene action is the pore formation model, in which polyenes and ergosterol interact to form an ion channel-like complex that leaks ions and small organic molecules from the cell, eventually leading to cell death [19]. Based on the amphipathic properties of polyenes, they would orientate in the plasma membrane with their hydrophobic polyene “tail” interacting with ergosterol, directed to the inner lipid environment of the membrane, while the hydrophilic polyol portion would form an aqueous channel, as illustrated in Figure 3A,B. Intermolecular hydrogen bonds between amino and carboxyl groups of the hydrophilic “heads” of neighboring polyene molecules further stabilize this channel [19]. Neutron diffraction studies have confirmed that such an architecture can exist when AmB interacts with ergosterol [44]. Typically, 4 to 12 polyene monomers would form a pore [39]. As the length of AmB is almost equal to the length of a membrane phospholipid on average, two types of channels can be made: a full pore consisting of two ring complexes of polyenes (see Figure 3A) and a ”half-pore”, containing only one polyene ring (see Figure 3B). Both types essentially have the same structure but the latter would induce a conformational thinning of the lipid bilayer [19].

Which pore is formed would primarily depend on the polyene, and the composition and thus thickness of the membrane [19]. For example, AmB would primarily form half-pores in a membrane mainly composed of dimyristoylphosphatidylcholine (DMPC) [45]. Pores are only formed after a certain threshold of polyene molecules in the membrane is reached. Below this threshold, aggregate complexes termed “non-aqueous pores” or “cation-selective pores” can increase the membrane permeability to monovalent cations, while true pores and half-pores can also transport larger nonelectrolyte molecules [46]. This threshold is significantly lower (by factor 5–10) in ergosterol containing membranes, compared to cholesterol containing membranes [46]. Moreover, patch clamp experiments with artificial AmB channels have shown that the ion transport occurs faster (a shorter channel ”dwell time”) in ergosterol containing membranes compared to cholesterol containing membranes [47], showcasing their antifungal specificity. The diameter of the pore determines the selectivity of transport or “leakage” out of the cell and depends on the type and concentration of polyene, while sterol type and sterol concentration have minor influences [19,39,48]. This is shown in the study by Yang et al., in which the channel diameter increases 100-fold when the concentration of AmB multiplies with factor 40 in an ergosterol-rich membrane [48]. In general, AmB forms relatively wide pores (approximately 0.46 nm) and can transport molecules as big as sucrose, while nystatin forms smaller pores (approximately 0.36 nm) [19].

### 3.3. Surface Adsorption and Sterol Sponge Models

The second and third models for polyene mode of action both hypothesize that, by adsorption or extraction of ergosterol from the membrane, the phospholipid membrane is destabilized, and essential cellular processes such as endocytosis and regulation of membrane protein function are disturbed [19]. Polyenes could adsorb ergosterol molecules to the “surface” of the phospholipid bilayer as illustrated in Figure 3C, termed the ”surface adsorption model” [33,49]. In light of this theory, Anderson and colleagues [32] conducted a series of NMR studies to determine the localization and structure of AmB interacting with ergosterol, and they observed that these complexes are not (always) inserted in the membrane and can form extra-membranous aggregates. They suggested that in such a mechanism, large aggregates of parallelly positioned AmB molecules can form at the membrane, functioning as a “sterol sponge” [32] as illustrated in Figure 3D. Extracting ergosterol from the membrane in such a “sponge” would perturbate a vast array of ergosterol-dependent cellular processes, many of which governed by membrane proteins that directly bind to ergosterol. This might also explain why resistance to polyenes is rarely observed, as in resistant cells an alternative membrane sterol such as lanosterol (a precursor of ergosterol) will probably malfunction in these processes and reduce the fitness and pathogenicity of the cell [32]. Anderson et al. [32] also suggest that the extraction of cholesterol by large extra membranous aggregates of AmB is the primary cause of toxicity of this drug towards mammalian cells and thus, optimizing the binding affinity of AmB derivatives to ergosterol could significantly improve its therapeutic efficacy. Some remarks have to be made regarding the sterol sponge model proposed by Anderson et al. [32]. First, the ergosterol-to-lipid ratio used in their experimental set-up is different from the one observed in natural systems [39]. As the ergosterol-to-lipid ratio is essential regarding the polyene susceptibility of fungi, this might play a vital role. Secondly, it has been proven that, in a cholesterol-saturated environment such as the mammalian cell membrane, the thermodynamical balance between ergosterol-AmB vs. cholesterol-AmB would shift towards the latter, meaning that the “sterol sponge” would be saturated with cholesterol rather than ergosterol. A third argument against this model is that fungi have a rigid hydrophilic cell wall composed of polymers of chitin that might prevent the passage of hydrophobic ergosterol and so the formation of super-aggregates or “sponges” outside the cell wall [39].

Ion channel formation, small membrane-spanning aggregates and large extra-membranous aggregates may exist at the same time [32], although the chemical structure of the polyene probably influences the primary mode of action. Several studies have shown that the elimination of the C35 hydroxyl group of AmB does not alter the sterol-adsorption capacity and cytotoxic effect of the molecule but eliminates its pore-forming capacity [33,50]. It was suggested that the ability to form pores depends on the dimensions of the polyene macrolactone ring and, therefore, polyenes can be divided into non-pore forming polyenes (e.g., natamycin) and the pore-forming polyenes (e.g., AmB and nystatin) [19].

### 3.4. Other Proposed Modes of Action

Several observations point towards oxidative damage as an additional mode of action of AmB [51,52]. One example is the rescue effect of hypoxia, exogenous catalase and super oxide dismutase (SOD) during AmB treatment of *C. albicans* without hindering AmB induced K^+^-leakage [52]. Another example is the enhanced resistance to oxidative damage by H_2_O_2_ of AmB resistant *C. albicans* strains [51]. Currently, several studies have provided evidence that polyenes can induce oxidative stress and cause DNA damage, protein carbonylation and lipid peroxidation, eventually leading to or contributing to cell death in fungi [53]. Moreover, metabolomic analysis of *C. albicans* exposed to AmB pointed out that AmB induced cell death was attenuated through increased production of polyamines such as putrescine, spermidine and spermine which have a role in scavenging reactive oxygen species (ROS) [54]. This is supported by gene expression analysis of *C. albicans* exposed to AmB, showing an increased expression of stress-related genes besides genes involved in membrane sterol homeostasis [55]. In *Cryptococcus neoformans*, it was shown that, after addition of AmB, cells become metabolically inactive and encounter a strong oxidative burst suggested to contribute to AmB induced cell death apart from membrane interactions and pore formation [35]. How this oxidative stress is exactly caused is still not clear, although it was suggested that polyene binding to the membrane triggers this response that leads to an apoptotic such as phenotype that includes ROS production or that, since AmB auto-oxidizes and forms free radicals [56], the antifungal itself causes oxidative stress [35]. In the latter model, the oxidative stress effect of polyenes would be distinct from its membrane permeabilization properties, although the free radicals produced would affect the membrane itself through lipid peroxidation [35].

## 4. Drug Resistance to Polyenes

**Figure 4:**
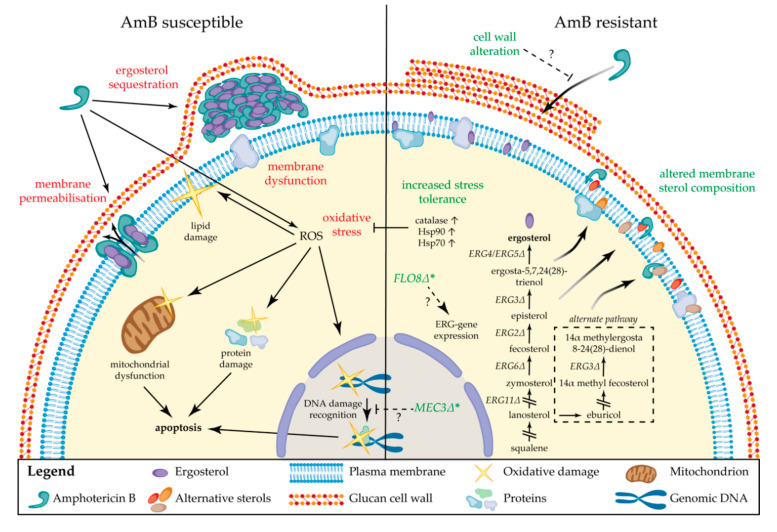
Schematic overview of the amphotericin B modes of action and drug resistance mechanisms in fungal cells. * association exists but has not been validated. See text for explanation. Gene names with delta symbol indicate mutations and not full deletions.

Although polyenes have been used for many decades [5], polyene resistance is still rare compared to resistance to other antifungal drugs [20]. One explanation for this is the fact that it is often associated with severe fitness trade-offs [20]. Another hypothesis is that, compared to most other antifungals, polyenes target a major cell membrane component instead of an essential enzyme. Nevertheless, several mechanisms of resistance have been proposed (Figure 4) as described in Section 4.1. Molecular Mechanisms of Resistance, and several pathogenic fungi species are known to possess intrinsic resistance or are able to acquire resistance to polyenes in the clinic, as described in Section 4.2. Epidemiology of Polyene Resistance.

### 4.1. Molecular Mechanisms of Resistance

The most common mechanism of acquired AmB resistance is attributed to alterations in the sterol composition of the fungal cell membrane [57,58]. Several mutations in genes of the ergosterol biosynthesis pathway (*ERG* genes) have been associated with this mechanism. In *C. albicans*, the loss of function of *ERG11* and *ERG3* genes (lanosterol 14α-demethylase and C-5 sterol desaturase, respectively) leads to the exchange of ergosterol for alternate sterols such as lanosterol, eburicol and 4,14-dimethyl-zymosterol in the membrane [20,58]. In other reports, AmB resistance in *C. albicans* is associated with a substitution in *ERG11* and loss of function of *ERG5* (C-22 sterol desaturase), again associated with an alternate membrane sterol composition [20,59]. In other *Candida* spp., inactivation of *ERG6* (C-24 sterol methyl-transferase) [60,61] and *ERG2* (C-8 sterol isomerase) [61] were found to have a similar effect. A mutation in *ERG2* resulting in its inactivation, is one of the only described mechanisms of AmB resistance in *C. neoformans* [62]. Nevertheless, high AmB resistance (MIC 20 µg/mL) without significant alterations in ergosterol biosynthesis have been reported in experimentally evolved *C. neoformans*, suggesting that sterol-independent mechanisms of AmB resistance also exist in *Cryptococcus* spp. [63]. Isolates of the *Candida haemulonii* species complex (*C. haemulonii*, *C. haemulonii* var. *vulnera* and *C. duobushemulonii*), known for their increased AmB resistance, have been shown to possess a cell membrane with an altered sterol profile similar to AmB resistant species with *ERG11*, *ERG3 ERG2* and *ERG6* mutations [64]. Given the severe fitness deficits of mutations in the ergosterol biosynthesis pathway [20], AmB resistance is rarely found in combination with resistance to other antifungal drugs, although certain polyene resistance inducing *ERG* mutations can result in cross resistance to azoles [58,65,66]. A well-known example of this occurs upon inactivation of the target of azoles *ERG11* and *ERG3*, resulting in the abrogation of the biosynthesis of ergosterol and inhibition of the synthesis of the toxic sterol 14-methyl-3,6-diol, respectively [58].

The growing interest in the role of oxidative stress in the fungicidal effects of AmB has led to an increase in research concerning stress related mechanisms of resistance. It is hypothesized that a reduction in polyene induced oxidative stress might allow the cell to better tolerate AmB exposure [53,67]. In intrinsically AmB resistant fungal pathogens such as *A. terreus*, this mechanism of resistance appears to be more important than an altered membrane sterol composition [67]. Compared to AmB susceptible *Aspergillus fumigatus*, intrinsically resistant *A. terreus* shows only a slight increase in membrane ergosterol levels, but a significant increase in catalase levels [68]. As research shows, a catalase-based mechanism of resistance counteracting AmB-induced oxidative stress that would otherwise result in cell death, is likely [35,51,52,53]. Catalase activity might break down the harmful ROS produced during AmB treatment and so protect the cell [69]. Further evidence supporting this hypothesis was given by Blatzer et al. [70], who found a severe induction of mitochondrial ROS generation upon AmB exposure in rare susceptible isolates of *A. terreus*, compared to resistant isolates [67]. Another mechanism of stress tolerance and/or resistance upon AmB exposure is likely governed by the molecular chaperone heat shock protein 90 (Hsp90), a highly connected, central factor in cellular stress responses that possibly potentiates the acquisition of resistance by regulating a myriad of client proteins [71,72]. Cowen et al. [72] hypothesize the involvement of Hsp90 in the emergence and/or maintenance of resistance via different possible mechanisms: (i) the disruption of Hsp90 might allow the persistence of new genetic variations, (ii) active Hsp90 might stabilize mutated cell regulators that have the ability to induce resistance but are prone to misfolding or (iii) Hsp90 might chaperone unmutated regulators of cell signaling (e.g., calcineurin), thereby allowing the development of adaptive phenotypes [72]. The connection between Hsp90 and antifungal resistance can be investigated through the use of Hsp90 inhibitors such as geldanamycin and radicicol [73,74]. In fluconazole resistant strains, modest inhibition of Hsp90 leads to abrogation of resistance but has no effect on cell growth [72]. Interestingly, inhibition of Hsp90 in AmB resistant cells does lead to inhibition of growth, even in the absence of AmB [20]. Blatzer et al. [75] found a correlation between AmB resistance in *A. terreus* and another family of chaperones: the Hsp70 family. They identified higher basal levels of expression of several Hsp70 genes in resistant strains and observed an instant activation of the majority of Hsp70 genes in the resistant *A. terreus* isolates upon exposure to AmB. Inhibition of Hsp70 only had limited effects on AmB susceptible *A. terreus* isolates, while it led to a considerable increase in susceptibility for resistant isolates [75]. Overall, these observations indicate a possibly essential role for the molecular chaperones of the Hsp90 and Hsp70 family in the acquisition and/or maintenance of AmB resistance [20,75].

In addition to alterations of the membrane sterol composition and regulation of oxidative stress, some reports show a correlation between AmB resistance and alterations of the fungal cell wall. In 1999, Seo et al. [76] acquired an experimentally evolved *Aspergillus flavus* isolate that was able to grow in concentrations of up to 100 µg/mL AmB [76]. An alteration of the cell wall was suggested to be an important mechanism contributing to this resistance. The main change in the fungal cell wall composition was reported to be an increase in the 1,3-α-glucan fraction [76]. More recently, an AmB resistant *C. tropicalis* isolate was also found to have an enlarged cell wall with increased levels of 1,3-β-glucan [77]. Although the increased glucan production could be the result of regulatory mechanisms affected by AmB exposure or resistance [76], another hypothesis is that the glucan molecules can physically inhibit the penetration of AmB through the cell wall [76]. In addition to increased AmB resistance, the cells with an enlarged cell wall also elicited a different immune response compared to susceptible cells. Mesa-Arango et al. [77] suggest that the increased 1,3-β-glucan is proportional to the strength of the immune response and contributes to prolonged survival of resistant clones since an exaggerated proinflammatory response can reduce the efficiency of immune clearance of a systemic infection [77,78].

The active efflux of drugs, a common mechanism of resistance for azole drugs, does not appear to play a role for polyene resistance. Although one report indicates an increased AmB tolerance in *C. albicans* upon the overexpression of drug efflux pump Cdr1 [79], this was later contradicted by Niimi et al. [80], who did not find a correlation between Cdr1 overexpression and polyene resistance.

Although multiple mechanisms of resistance have been proposed, the molecular mechanisms driving a reduced polyene susceptibility are not always clear. This is showcased by the emergent *C. auris*, in which an AmB resistance prevalence of up to 30% [81,82,83] has been found among clinical isolates, but still sequencing efforts cannot identify driving mutations. Differential expression of *ERG* genes might be involved, as Munoz et al. [84] showed an upregulation of *ERG13*, *ERG6*, *ERG2* and *ERG1* upon AmB exposure in AmB resistant *C. auris* isolates, compared to susceptible strains [84]. Escandon and colleagues [18] sequenced AmB resistant *C. auris* strains of Colombia and identified a mutation in a gene encoding a homolog of *C. albicans FLO8*. Flo8 is a transcription factor that has multiple downstream effectors, and it has been proven to positively regulate *ERG11* expression in yeast [85]. A recent study by Carolus et al. [86] also identified a mutation in *FLO8* in an AmB resistant *C. auris* strain that was experimentally evolved. Although this mutation did not significantly alter the AmB MIC, it raises questions regarding the link between *FLO8* and AmB resistance in *C. auris*. The same strain from Carolus et al. [86] also acquired nonsense mutations in both *ERG11* and *ERG3*, significantly decreasing the AmB susceptibility with cross resistance towards azoles [86]. Additionally, a mutation was found in a gene predicted to encode the DNA damage checkpoint protein Mec3 [86], part of the Rad17p-Mec3p-Ddc1p sliding clamp [87]. As AmB evokes oxidative damage to DNA that leads to apoptosis [53], DNA damage checkpoint proteins might be involved in AmB resistance, although this link has not been confirmed. Due to these and other loose ends, further research into the mechanisms of polyene resistance, especially in the context of pan resistance as found in *C. auris* [81,88,89], *Fusarium* [90,91,92] and *Scedosporium* spp. [93,94,95,96], is highly needed [97,98].

### 4.2. Epidemiology of Polyene Resistance

AmB resistance occurs in both yeasts and molds [99,100,101,102,103,104]. *C. albicans*, the most common cause of human candidiasis, rarely shows resistance to polyenes, although resistant strains have been found in antifungal susceptibility surveillance studies [21,101,105,106]. Similarly, *Candida glabrata*, *Candida parapsilosis*, *Candida guilliermondii*, *Candida kefyr* and *Candida dubliensis* are generally considered to be susceptible to AmB, with most surveillance studies reporting an AmB susceptibility rate close to 100% [21,100,101,105,106,107,108,109,110,111,112]. *Candida tropicalis* [102,107,109,111] and *Candida krusei* [21,102,105,106] are often found to be AmB susceptible, but some reports show AmB resistance, with higher rates in *C. krusei* [107,111,112] compared to *C. tropicalis* [112]. Reports on AmB resistance in *Candida lusitaniae* range from relatively frequent [113,114,115] to similar resistance rates compared to other *Candida* spp. [102,116,117,118]. In comparison to to the previously mentioned *Candida* spp., *C. auris* [81,88] and species of the *C. haemulonii* complex [119,120,121,122] are frequently observed to be AmB resistant. These emergent *Candida* spp. are closely related and show strong tendencies towards intrinsic and/or acquired resistance to AmB [81,88,119,123]. For *C. auris*, AmB resistance rates range from 8% [88] up to 23% [81]. Among the *C. haemulonii* species complex, resistance rates are even higher, with one study reporting a high AmB MIC for 12 out of 28 isolates [120]. Other reports [119,121,122] confirm a high rate of polyene resistance in *C. haemulonii complex* spp., suggesting an intrinsic resistance to AmB. In general, *C. duobushaemulonii* exhibits the highest MICs for AmB, often reaching twice the MIC of *C. haemulonii* [119,120,121,122]. *C. neoformans* and *C. gattii* are the most common, pathogenic non-*Candida* yeasts [124,125], but AmB resistance in *Cryptococcus* spp. is rare, with resistance rates varying from 0% to 5.8% for *C. neoformans* and *C. gattii*, respectively [126,127,128,129,130,131,132,133]. Regarding species of the *Trichosporon* genus, *Trichosporon asahii* (formerly *T. beigelii*) is most frequently found to infect humans [134,135]. *T. asahii* shows the ability to acquire extensive resistance to AmB, reaching resistance rates of over 50% [136,137,138,139,140,141]. Other pathogenic yeasts not mentioned here are less clinically relevant and/or are generally susceptible to AmB [142].

Among pathogenic molds, *Aspergillus* spp. are well known for their extensive AmB resistance. For *A. fumigatus*, the most common cause of invasive aspergillosis [143,144,145,146], recent studies have shown the ability to efficiently acquire AmB resistance with rates ranging from 43% [144] to 96.4% [146]. Widespread AmB resistance is also found among other common *Aspergillus* spp. such as *A. flavus* (resistance rate ranges from 12.5% [147] up to 87% [144]) and intrinsic AmB resistance is well recognized in *A. terreus* [67]. Furthermore, intrinsically resistant isolates have been suggested for several other *Aspergillus* spp. such as *A. flavus* [145,148] and *Aspergillus lentulus* [145,149]. It should be noted that some studies [150] report a low incidence of AmB resistance in *Aspergillus* spp. Baddley et al. [151] report, for example, low AmB MICs for 93.3% of the investigated *Aspergillus* spp. isolates with increased MICs only in *A. terreus* [151]. Other filamentous fungi such as *Scedosporium apiospermum* and *Lomentospora prolificans* (formerly *Scedosporium prolificans*) have also been identified as intrinsically AmB resistant and, therefore, often require complex antifungal treatments [93,94,95,152,153]. Additionally, some *Fusarium* spp. have been reported as intrinsically resistant to AmB [90,91,92,154]. While AmB is usually recommended for treating invasive fusariosis [13], *Fusarium solani*¸ a prevalent *Fusarium* spp. often shows increased AmB MICs [155,156]. Other members of the *Fusarium* genus are generally susceptible to AmB. *Sporothrix schenkii* is usually observed to be susceptible to AmB although some resistant isolates have been found [157]. Furthermore, several reports indicate increased AmB MICs for the hyphal form of this pathogen compared to the yeast form [158,159,160], complicating clinical interpretation [158]. *Histoplasma capsulatum* exhibits very low MICs for AmB, making it a preferred treatment for this pathogenic mold [161,162]. Although AmB resistance is rare for isolates of Mucorales [163,164,165], some *Rhizopus* and *Cunninghamella* strains show decreased AmB susceptibility [103,166], with one report showing decreased AmB susceptibility in 3 out of 19 and 5 out of 8 isolates tested, respectively [167].

A major challenge in investigating the epidemiology of polyene resistance is the lack of clear susceptibility breakpoints for most fungal pathogens. Park et al. [168] showed that the commonly used breakpoint of 2 µg/mL for *Candida* spp. does not always correlate with the clinical outcome of AmB treatment. Rex et al. [169] showed similar results and even observed an inversely proportional trend, where lower MIC values were associated with increased failure of treatment in the clinic. Nonetheless, some studies report a positive correlation between AmB MIC and treatment success [170,171]. For most fungal species, no clinical breakpoints have been determined, thus forcing researchers to resort to epidemiological cutoff values to distinguish resistant from susceptible isolates. These conflicting observations complicate and question the interpretation of resistance epidemiology and its clinical significance.

## 5. Conclusions

Although discovered over 70 years ago, and despite their inherent toxic nature, polyene antifungals are still part of the first-line treatment for various types of invasive fungal infections. This showcases both the predicament of the antifungal drug discovery pipeline and the potency of polyenes as antifungal drugs. Polyenes have been used in the clinic over several decennia but still, debates are ongoing regarding their modes of action, and the mechanisms of resistance are arguably the least understood compared to other commonly used antifungal drugs such as azoles and echinocandins. Emergent polyene resistant pathogens such as *C. auris,* for which hardly any reports on resistance mechanisms exist despite a high incidence of resistance in the clinic, urge to fill those knowledge gaps. A multitude of recent reports show the interest in further development of AmB formulations, including oral drug development, to facilitate use and increase treatment efficacy. Especially due to their broad spectrum, rare incidence and relatively weak potency of resistance development, polyenes could still be described as a ”diamond in the rough” in the current antifungal drug resistance crisis. Nonetheless, for polyenes to be on the forefront of this battle, further research into their mode of action, drug resistance and clinical application is key.
